# Electrochemical Synthesis of Bismuth Particles: Tuning Particle Shape through Substrate Type within a Narrow Potential Window

**DOI:** 10.3390/ma10010043

**Published:** 2017-01-06

**Authors:** Doga Bilican, Jordina Fornell, Jordi Sort, Eva Pellicer

**Affiliations:** 1 Departament de Física, Facultat de Ciències, Universitat Autònoma de Barcelona, E-08193 Bellaterra, Spain; doga.bilican@uab.cat (D.B.); jordina.fornell@uab.cat (J.F.); jordi.sort@uab.cat (J.S.); 2 Institució Catalana de Recerca i Estudis Avançats (ICREA), Pg. Lluís Companys 23, E-08010 Barcelona, Spain

**Keywords:** bismuth, electrodeposition, X-ray diffraction

## Abstract

Bismuth (Bi) electrodeposition was studied on Si/Ti/Au, FTO-, and ITO-coated glasses from acidic nitrate solutions with and without gluconate within a narrow potential window (ΔE = 80 mV). This potential range was sufficient to observe a change in particle shape, from polyhedrons (including hexagons) to dendrites, the trend being slightly different depending on substrate activity. In all cases, though, the formation of dendrites was favoured as the applied potential was made more negative. Bi particles were more uniformly distributed over the substrate when sodium gluconate was added to the electrolyte. X-ray diffraction analyses of dendrites grown at −0.28 V indicated that they exhibit the rhombohedral phase of Bi and are predominantly oriented along the (003) plane. This orientation is exacerbated at the lowest applied potential (−0.20 V vs. Ag|AgCl) on glass/ITO substrate, for which completed and truncated hexagons are observed from the top view scanning electron microscopy images.

## 1. Introduction

Electrodeposition is a surface treatment method which allows the growth of films, micro- and nanostructures with a simple and relatively low-cost set-up. By changing the parameters, such as applied potential or current density, composition of the electrolyte, and the substrate type, it is possible to obtain deposits with different properties and functions. Electrodeposition of semi-metal bismuth has attracted much attention due to its unique physical and chemical properties, which make it a choice for a wide range of applications in different areas, including magnetoresistance [1,2], thermoelectricity [3], superhydrophobic surfaces [4], or electroanalysis [5,6,7]. The fact that bismuth is a chemically non-hazardous metal makes it a preferable option for environmentally sustainable processes, such as providing an alternative to mercury electrodes [5].

It has been shown that bismuth can be electrodeposited in different configurations, ranging from dense or particulated films [8] to sparse particles, such as polyhedrons (most typically hexagons) [9], dendrites, rods, or fern-like particles by properly adjusting the electrochemical parameters. Continuous relatively thick films, micro- and nanodendrites, and hexagons made of bismuth have been typically reported from aqueous nitrate solutions. In the past decade, Jian et al. observed that the dendrite-like morphology of submicrometer bismuth particles deposited from Bi(NO_3_)_3_·5H_2_O dissolved in diluted nitric acid could be tuned, to some extent, through the addition of ethylenediaminetetraacetic acid (EDTA) to the bath and the substrate type (Pt, Al, and indium tin oxide (ITO)) [10]. A few years later, Yang and Hu studied the first stages of bismuth electrodeposition on glassy carbon electrodes (GCE) from 1 M HNO_3_ + 5–20 mM Bi(NO_3_)_3_·5H_2_O and produced small crystallites over GCE at cathodic potentials between −100 and −300 mV vs. Ag|AgCl (3 M KCl) and deposition times ranging from 10 to 350 s [11]. Sandnes et al. studied the electrodeposition of Bi from electrolytes containing different concentrations of Bi(NO_3_)_3_ dissolved in 1 M HNO_3_ [12]. The authors concluded that Au electrodes were the most suitable ones to examine the reduction of Bi^3+^ in acidic nitrate solutions since electro-reduction of nitrate is very slow and marginally detectable. Bulk deposition of Bi was observed ca. −0.5 V vs. SSE (i.e., at −0.05 V vs. Ag|AgCl (3 M KCl)) over a pre-deposited Bi grown by underpotential deposition (UPD). Compact, relatively thick films (1.0–15 μm thick) were characterized morphologically and structurally. More recently, fern-shaped Bi dendrites were grown on GCE using a two-step potentiostatic deposition scheme consisting of applying −0.5 V for 200 s followed by −0.8 V for 1000 s. In the second deposition step, hydrogen co-evolution took place [13].

Compared to compact films, submicron- and nanostructures are known to possess larger surface-to-volume ratios and, therefore, their production attracts a great deal of interest for surface-sensitive applications like heterogeneous catalysis, gas storage, and sensing. Most frequently, low populated surfaces consisting of small nuclei/crystals relatively far away from each other are reported in the literature [9]. However, in some cases, densely populated electrodes featuring a large density of, though not yet coalesced, particles can be interesting. In this work, the positive effect of gluconate additive on electrode coverage is addressed. Whilst deposition of Bi from citric acid, EDTA, and polyethylene glycol (PEG) baths has been exploited in the past [8,10], gluconate-containing acidic baths have not been explored yet.

In this work, micrometer- and submicrometer-sized bismuth particles are electrodeposited onto Si/Ti/Au, ITO-coated, and fluorine-doped tin oxide (FTO)-coated glass substrates. The beneficial effect of gluconate additives on electrode coverage uniformity is reported. We show that particle shape and size can be tuned within a very narrow potential window. The potential at which the transition from polyhedral particles to dendrites occurs is substrate-dependent.

## 2. Results and Discussion

### 2.1. Cyclic Voltammetry (CV) Studies

**Figure 1:**
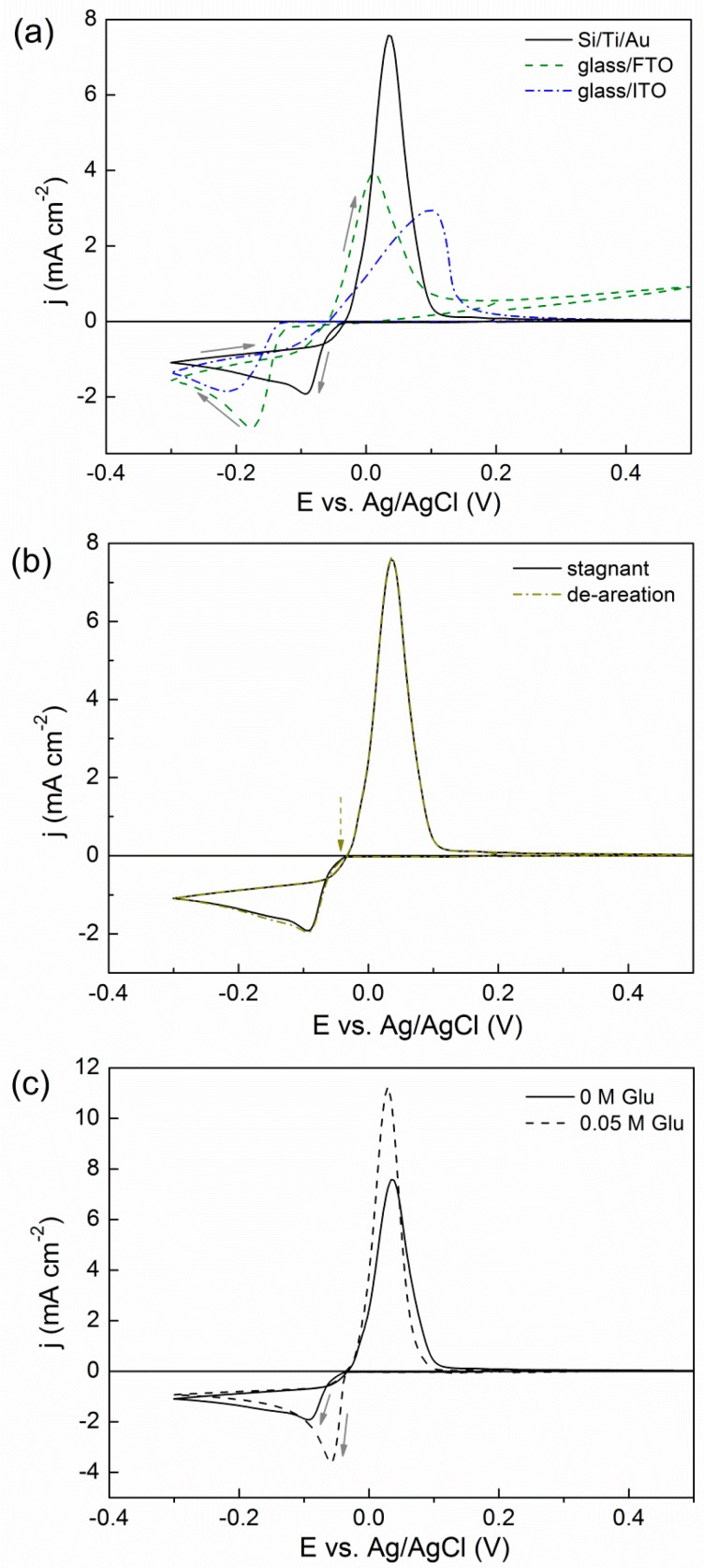
CV curves recorded at 50 mV·s^−1^ from 5 mM Bi(NO_3_)_3_·5H_2_O + 1.5 M HNO_3_ + *x* M NaC_6_H_11_O_7_ with (**a**) *x* = 0 onto different substrates in stagnant conditions; (**b**) *x* = 0 onto Si/Ti/Au in stagnant and Ar-bubbling conditions (initiated at the crossover toward positive potentials as indicated by the dashed arrow); and (**c**) *x* = 0 and *x* = 0.05 onto Si/Ti/Au in stagnant conditions.

The CV responses from 5 mM Bi(NO_3_)_3_·5H_2_O + 1.5 M HNO_3_ electrolyte onto Si/Ti/Au, glass/FTO, and glass/ITO substrates are shown in Figure 1a. Due to the different activity of the substrates, the onset of reduction is different, this being shifted towards more negative potentials within the series of Si/Ti/Au > glass/FTO > glass/ITO. Deposition of bulk Bi took place from −40 mV on Au surface, in agreement with [12]. A diffusion-like peak was recorded in all cases. In the anodic scan, a single oxidation peak was recorded on all electrodes, as well, between −30 and 300 mV. 

Enhanced mass transfer provided by Ar bubbling through the electrolyte during the entire CV led to higher current densities in the cathodic scan and, concomitantly, higher oxidation currents in the anodic scan (not shown). In order to confirm that H_2_ co-evolution was not involved (or marginally involved) at the screened potentials in spite of the low pH, CVs including initiation of Ar purging at the positive sweep were carried out and compared to those obtained under stagnant conditions (Figure 1b). The oxidation current did not decrease when the Ar flux was switched on at the cross-over. Therefore, almost no H_2_ bubbles had been previously adsorbed onto the cathode in the negative sweep. If that would have been the case, detachment of hydrogen bubbles from the surface due to Ar purging would have caused a significant decrease of the oxidation current.

The addition of gluconate to the electrolyte did not delay the onset of deposition but caused a shift toward more positive potentials (Figure 1c). This effect was more pronounced on Si/Ti/Au substrate than on glass/FTO and glass/ITO substrates. At the working pH, gluconate protonation takes place. Moreover, it has been reported that the protonation of gluconate is coupled with the lactonization of gluconic acid [14]. The ability of gluconic acid to bind metal ions is rather weak in acidic conditions [15] and this can explain why the onset of deposition is not shifted toward more negative potentials.

### 2.2. Potentiostatic Deposition and Morphological Characterization of Deposits

**Figure 2:**
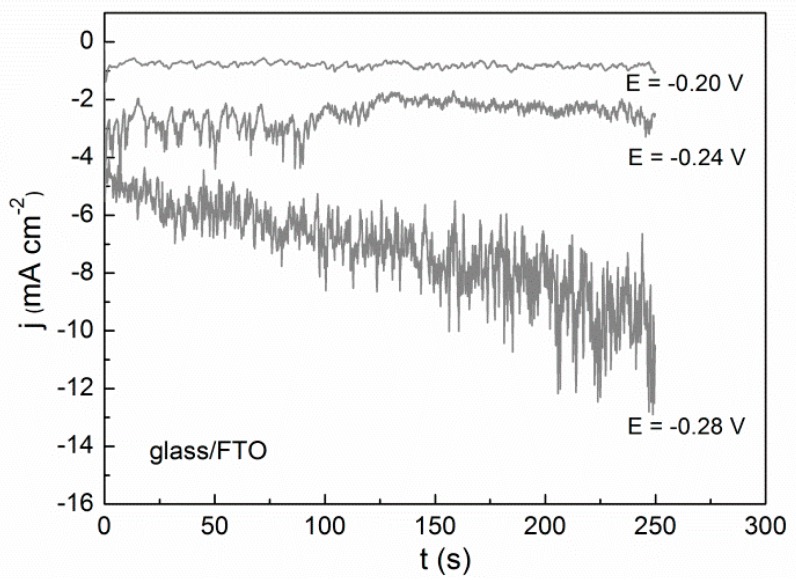
Current density-time (j–t) curves recorded from 5 mM Bi(NO_3_)_3_·5H_2_O + 1.5 M HNO_3_ onto glass/FTO electrode under Ar bubbling.

Micrometer- and submicrometer-sized Bi particles were grown by applying constant potentials between −0.20 and −0.28 V, i.e., within 80 mV interval, for deposition times ranging from 120 to 800 s. Noisy j–t curves were recorded (Figure 2) as argon was made to continuously bubble during deposition. For all three electrodes, higher current density was measured as the applied cathodic potential was made more negative.

The cathodic potential could not be extended beyond −0.3 V, especially on glass/ITO, as ITO suffered from instabilities at such acidic pH during cathodic polarization, mostly causing an abrupt increase of the resistance [16]. Moreover, since SnO_2_ in both FTO and ITO gets dissolved in the solution at high cathodic potentials in the low pH media, the bath cannot be reused further [17]. 

Immediately after deposition, deposits were rinsed in deionized water and dried in hot air. It was noticed that a N_2_ flux was not fully efficient to dry the deposits, i.e., in many cases the particles displayed an intricate morphology and remained wet. Meanwhile, if the samples were left to dry spontaneously, the remaining water droplets were capable of fully oxidizing the Bi film. 

**Figure 3:**
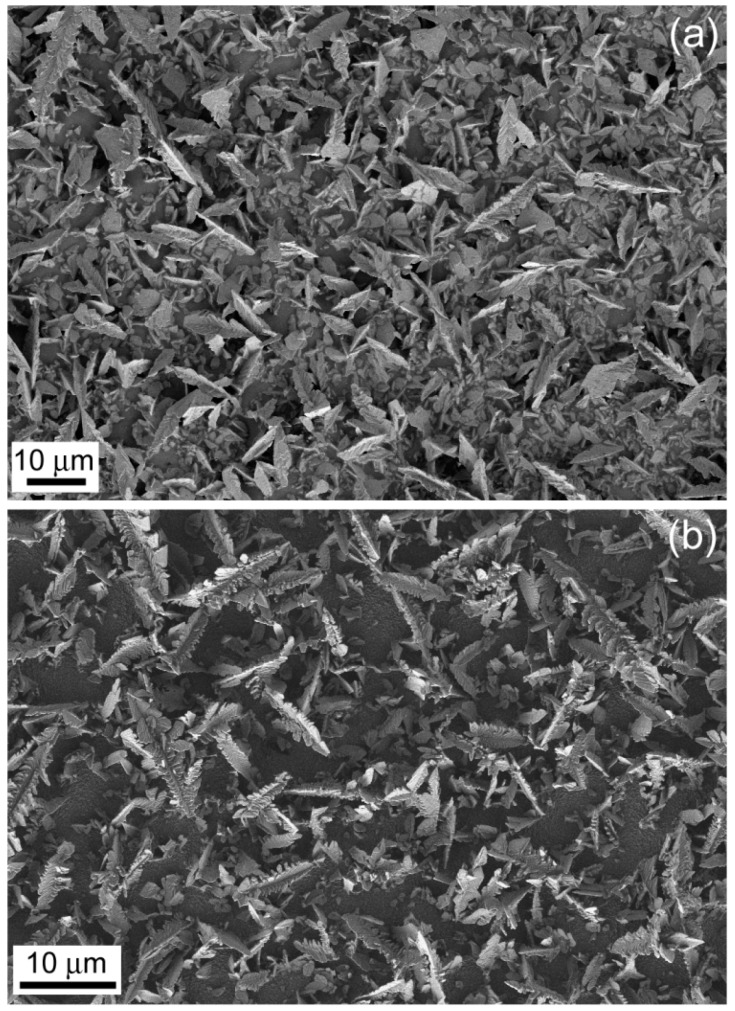
Scanning electron microscopy (SEM) images of Bi deposits obtained from a 5 mM Bi(NO_3_)_3_·5H_2_O + 1.5 M HNO_3_ bath under Ar bubbling at −0.28 V for 250 s on (**a**) glass/ITO and (**b**) glass/FTO.

**Figure 4:**
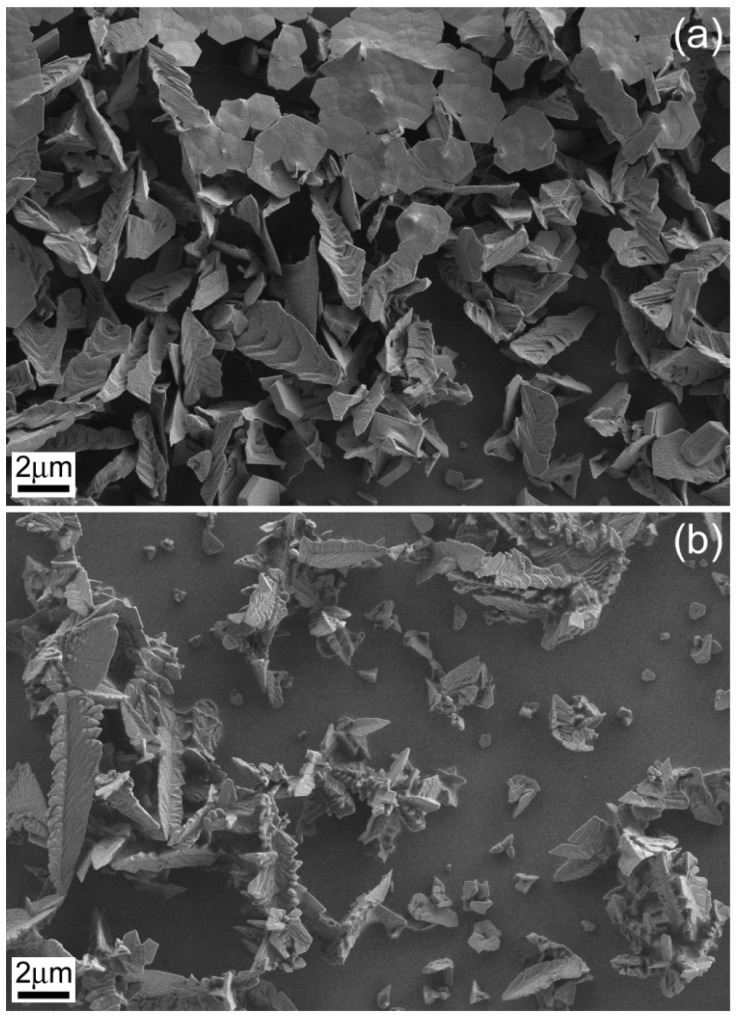
SEM images of Bi deposits obtained from a 5 mM Bi(NO_3_)_3_·5H_2_O + 1.5 M HNO_3_ bath under Ar bubbling at −0.28 V on ITO/glass for (**a**) 120 s and (**b**) 800 s.

The substrate type had an obvious impact on the particles’ shape. For example, sharper dendrites were observed on glass/FTO than on glass/ITO under identical deposition conditions (Figure 3). Deposition time also caused a change on particles’ morphology. Deposits obtained on glass/ITO at low deposition times displayed highly-faceted incipient dendrites which evolved toward a more branched type at higher deposition times (Figure 4). Nevertheless, deposition times longer than 250–300 s were not recommended since detachment of the as-deposited material was observed.

**Figure 5:**
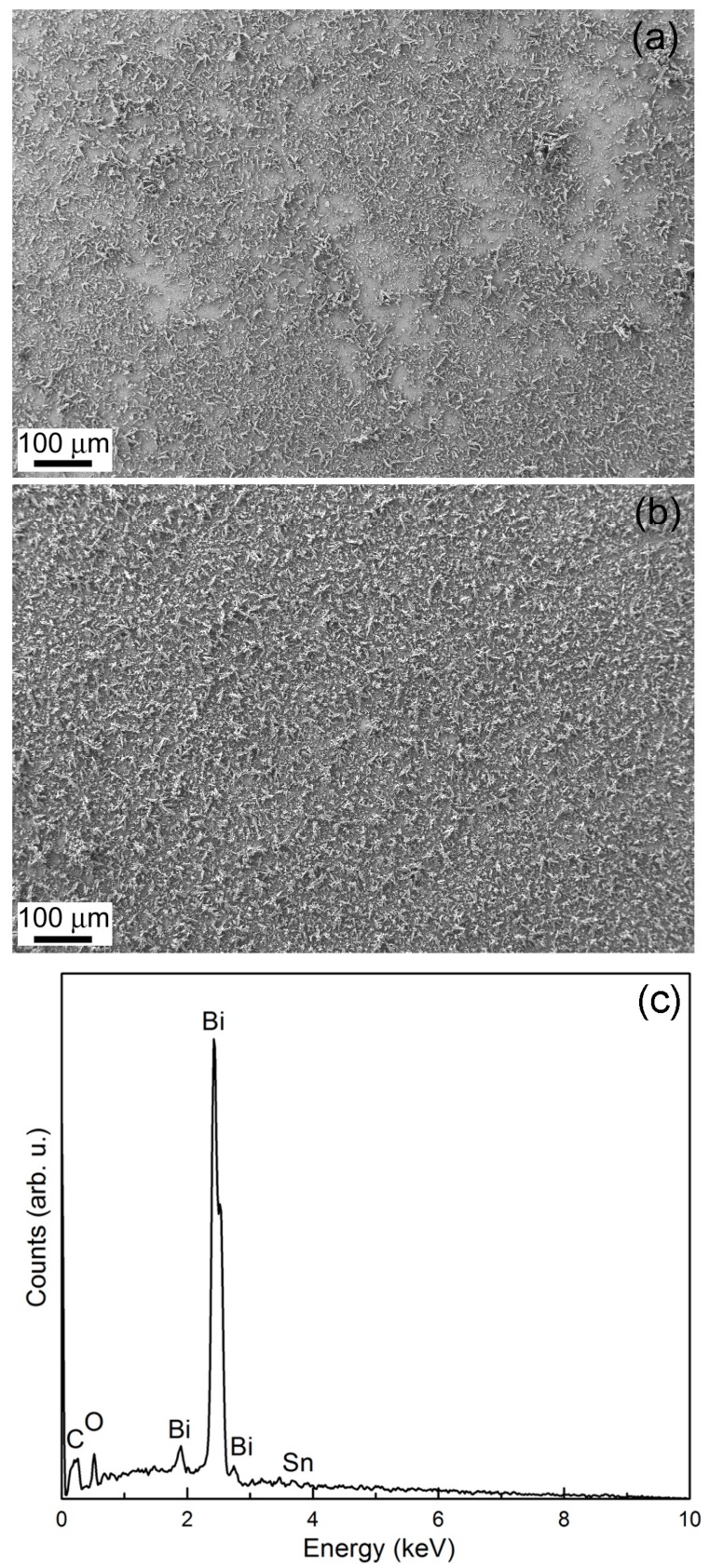
SEM images of Bi deposits synthesized at −0.24 V for 250 s on ITO/glass substrate from a bath with 5 mM Bi(NO_3_)_3_·5H_2_O + 1.5 M HNO_3_ + *x* M NaC_6_H_11_O_7_ for (**a**) *x* = 0 and (**b**) *x* = 0.05; (**c**) EDX pattern of the sample shown in (**b**).

The addition of gluconate to the electrolyte enabled the production of more uniformly covered substrates (Figure 5a,b). As aforementioned, gluconate mainly exists as gluconic acid in the bath. The complexing capacity of gluconic acid is much lower than that of the gluconate anion [18]. As a result, gluconic acid acts as a throwing power additive rather than as a complexing agent. A representative energy dispersive X-ray (EDX) pattern is shown in Figure 5c, indicating the presence of Bi along with a residual contribution of O and the peaks coming from the substrate. 

**Figure 6:**
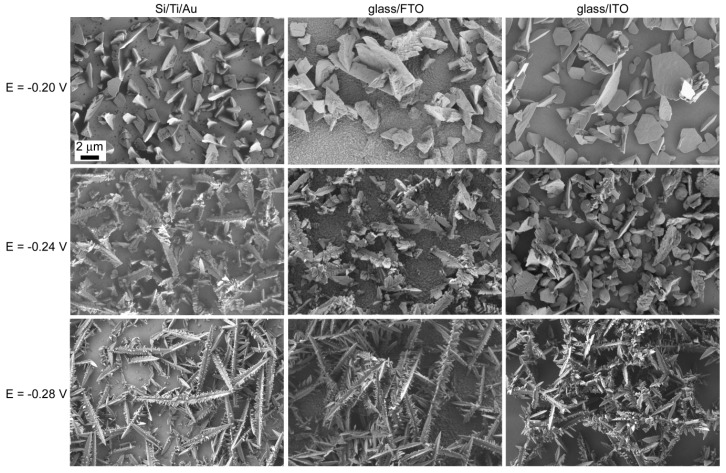
SEM images of bismuth electrodeposited at −0.20 V, −0.24 V, and −0.28 V for 250 s from a 5 mM Bi(NO_3_)_3_·5H_2_O + 1.5 M HNO_3_ + 0.05 M NaC_6_H_11_O_7_ bath on Si/Ti/Au, glass/FTO, and glass/ITO substrates. The scale bar is the same for all images.

Good adhesion was noticed on all substrates for deposition times up to 250 s. Applying longer durations caused detachments from the substrate, similar to the gluconate-free bath. Polyhedral particles and incipient dendrites were observed on glass/FTO at −0.20 V (Figure 6). Dendrites continued to form at −0.24 V and fully developed at −0.28 V. A transition from polyhedrons to dendrites was also observed on Si/Ti/Au, although the particles looked much more pointed (i.e., triangles) at the lowest potential applied. Meanwhile, both incomplete and perfect hexagons were mostly found on glass/ITO at −0.20 V. Dendrite formation started at −0.24 V and progressed at −0.28 V. Dendrites were sharper on glass/FTO and Si/Ti/Au substrates than on glass/ITO. It was, thus, clear that the particles’ morphology was dependent on substrate type. The cathodic potential was not further increased as this caused the dendrites to detach from the substrate, in agreement with previous observations [12]. This transition from polyhedrons toward dendrites with slightly decreasing the potential was previously reported on glass/ITO substrate from a less acidic electrolyte and shorter deposition times [19].

The nature of the substrate influences the coating structure within the initial electrodeposition stages. Surface characteristics including surface finish (roughness), conductivity, and structure (amorphous, single-crystal, polycrystalline), have a decisive impact on the nucleation rate. In our case, Au is a metal whilst both ITO and FTO are semiconductors. Therefore, island nucleation and growth might be affected by substrate conductivity to a great extent [20]. This is clear upon observing when dendrites are formed, a phenomenon which is somehow delayed on both ITO and FTO compared to Si/Ti/Au substrates. 

At −0.20 V, the polyhedrons formed on Si/Ti/Au had sizes varying from 1 to 4.5 µm. On glass/ITO, they had lengths between 1.4 and 7 µm and width of 1 µm. Meanwhile, polyhedrons with a width up to 2 µm formed on glass/FTO, accompanied with dendrites up to 4 µm in length and 2 µm in width. As aforementioned, well-shaped hexagons were only observed on glass/ITO. In this case, their size distribution became narrower as the potential was made more negative; namely, at −0.20 V they exhibited sizes ranging from 600 nm to 3 µm, whereas at −0.24 V 1 µm-sized hexagons were obtained. Concomitantly, dendrites with lengths up to 7.2 µm and widths up to 2 µm were seen on this substrate. Compared to glass/ITO, smaller dendrites formed on glass/FTO at −0.24 V, with lengths reaching up to 4 µm and widths of 600 nm. As the absolute value of the cathodic potential increased from −0.24 to −0.28 V, the lengths of the dendrites showed an increase on each substrate up to 10 µm and a decrease in their widths down to 600 nm.

### 2.3. X-ray Diffraction Analysis

**Figure 7:**
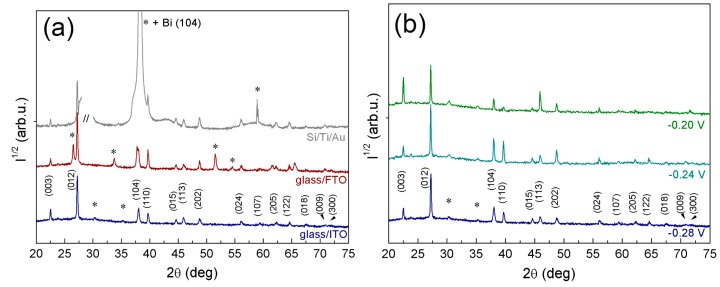
XRD patterns of Bi deposits obtained from 5 mM Bi(NO_3_)_3_·5H_2_O + 1.5 M HNO_3_ + 0.05 M NaC_6_H_11_O_7_ for 250 s (**a**) at −0.28 V on Si/Ti/Au, glass/FTO, and glass/ITO substrates and (**b**) on glass/ITO substrates at −0.20 V, −0.24 V, and −0.28 V. Peaks denoted with (*) belong to the substrate.

The collected X-ray diffraction (XRD) patterns indicated that single-phase deposits consisting of pure Bi crystallizing in the rhombohedral lattice (space group R-3̅m, classed into the hexagonal crystal system) were obtained irrespective of the applied potential and substrate type. In order to study the influence of the substrate on the microstructure, XRD patterns of Bi electrodeposited at −0.28 V on Si/Ti/Au, glass/FTO, and glass/ITO substrates are presented in Figure 7a. At first glance, slight differences on the relative intensity of peaks among the deposits, which is indicative of texture, can be detected. To point this out, intensity ratios between the different reflections (i.e., (003), (104), etc.) and the most intense one (012) are calculated in Table 1. According to the I_(hkl)_/I_(012)_ ratios obtained, Bi dendrites grown on Si/Ti/Au, glass/FTO, and glass/ITO substrates show a predominant orientation along the (003) plane (i.e., the plane parallel to the base of the rhombohedron). This orientation is exacerbated in the dendrites grown on the glass/ITO substrates. 

Lattice parameters calculated by Rietveld refinement, as well as crystallite sizes determined by profile fitting, are listed in Table 2. According to Rietveld refinement, the lattice parameters of rhombohedral Bi at an applied potential of −0.28 V are close to the theoretical ones (*a* = 4.546 Å and *c* = 11.862 Å) but slightly lower, indicating possible lattice distortion. For each diffraction pattern, the full width at half maximum (FWHM) intensity of each peak was measured and the crystallite size was determined using the Williamson-Hall plot. At −0.28 V, slightly larger crystallite sizes are obtained for Bi grown on glass/FTO substrate (~49 nm), while smaller crystallite sizes are obtained for that grown on Si/Ti/Au substrate (~37 nm). To evaluate the contribution of the deposition potential on the microstructural features of Bi, XRD of deposits grown on glass/ITO at the three potentials studied in this work was carried out. The obtained diffraction patterns are presented in Figure 7b and cell parameters and crystallite sizes listed in Table 2. According to the I_(hkl)_/I_(012)_ ratios (Table 1) the more textured Bi grows at −0.20 V. In this case, strongly preferred orientation of (003) planes is readily apparent; this is in agreement with the morphology observed by SEM, where completed and truncated hexagons are observed from the top view; on the opposite way, the intensity of several other reflections, such as the (104), (110), and (202), decreases. This result seems to be in line with the analysis carried out by Sandnes et al. [12] where the reflection of the (003) plane only appears at their lowest applied potential (−0.05 V vs. Ag|AgCl (3 M KCl)). An important reduction on crystallite size is also detected for the particles grown at −0.20 V. It is worth noticing that the cell parameters of the Bi deposited at −0.20 V and −0.24 V are closer to the theoretical values.

## 3. Materials and Methods

CV studies and potentiostatic electrodeposition of bismuth at room temperature on Si/Ti (10 nm)/Au (90 nm), FTO-coated glass (glass/FTO, ρ = 8 Ω/sq), and ITO-coated glass (glass/ITO, ρ = 8–12 Ω/sq) substrates were implemented in a three-electrode cell with a Pt wire as the counter electrode and a Ag|AgCl (3 M KCl) reference electrode (E = +0.210 NHE). Electrode arrangement and separation were the same in all experiments. Two different baths were used, one of which consisted of 5 mM Bi(NO_3_)_3_·5H_2_O and 1.5 M HNO_3_ and the other with 0.05 M NaC_6_H_11_O_7_ (sodium gluconate), in addition to the components mentioned. Argon was purged into the electrolyte both before and during the electrodeposition to avoid possible oxidation and favour mass transport. Prior to electrodeposition, Si/Ti/Au substrates were conditioned by cleaning with acetone, isopropanol and Milli-Q water and dried with nitrogen, whereas glass/FTO and glass/ITO substrates were cleaned with isopropanol and dried. The working area was between 0.25 and 0.56 cm^2^. After the electrodeposition step, the samples were rinsed in Milli-Q water and hot dried. All samples were stored in a desiccator.

Scanning electron microscopy (SEM) observations were done using a Merlin Zeiss microscope (Jena, Germany) operated at 2 kV. Energy dispersive X-ray (EDX) analyses were carried out at 15 kV using a detector coupled to the SEM microscope. Wide-angle XRD patterns were collected on a Philips X’Pert diffractometer (Almelo, The Netherlands) in the 20°–75° 2θ range using CuK_α_ radiation. Rietveld refinement using MAUD software [21] was carried out to determine the lattice parameters. Crystallite size was calculated by profile fitting using the Williamson-Hall plot option in the HighScore Plus software (PANalytical, Almelo, The Netherlands).

## 4. Conclusions

Finely-distributed micrometer- and submicrometer-sized Bi particles were obtained by electrodeposition from an aqueous solution containing nitric acid and sodium gluconate. A narrow window of deposition potentials and relatively short deposition time (250 s) to prevent the material from detachment was examined. The substrate type (metallic vs. oxide) greatly impacted both the shape and the size of the particles obtained at each applied potential. Differences in substrate conductivity likely play a key role in Bi particles’ shape. A transition from polyhedral-shaped particles, including hexagons towards dendrites, was most clearly observed on glass/ITO. Rhombohedral Bi with (003) predominant orientation and crystallite size below 50 nm was obtained at −0.28 V on all electrodes. The (003) orientation further increased when completed and truncated hexagons formed on the substrate, as was the case for the glass/ITO electrode.

## Figures and Tables

**Table 1:** Theoretical (JCPDS 01-085-1329) and experimental intensity ratios, the latter determined from the XRD patterns shown in Figure 7, for Bi deposits obtained on glass/FTO, Si/Ti/Au, and glass/ITO.

Plane	I_(hkl)_/I_(012)_ (%)					
	Theoretical	Glass/FTO (−0.28 V)	Si/Ti/Au (−0.28 V)	Glass/ITO (−0.28 V)	Glass/ITO (−0.24 V)	Glass/ITO (−0.20 V)
(003)	5	9	7	16	17	62
(012)	100	100	100	100	100	100
(104)	33	24	–	18	26	17
(110)	34	19	30	11	23	7
(202)	16	7	6	5	12	10

**Table 2:** Cell parameters and crystallite size determined from the XRD patterns shown in Figure 7 for Bi deposits obtained on glass/FTO, Si/Ti/Au, and glass/ITO.

Substrate	Potential (V)	Cell Parameter (Å)		Crystallite Size (Å)
		*a*	*c*	
glass/FTO	–0.28	4.54256 ± 2.9 × 10^−4^	11.84982 ± 1.7 × 10^−4^	486 ± 59
Si/Ti/Au	–0.28	4.54525 ± 2.1 × 10^−4^	11.84953 ± 1.5 × 10^−4^	369 ± 45
glass/ITO	–0.28	4.54161 ± 1.1 × 10^−4^	11.85492 ± 3.3 × 10^−4^	456 ± 52
glass/ITO	–0.24	4.54647 ± 1.7 × 10^−4^	11.86142 ± 8.8 × 10^−4^	770 ± 96
glass/ITO	–0.20	4.54818 ± 2.4 × 10^−4^	11.86089 ± 6.6 × 10^−4^	154 ± 62
